# BDNF plasma concentrations, cognitive test performances and lifetime suicide ideation in psychotic disorders: a secondary analysis

**DOI:** 10.1192/j.eurpsy.2024.166

**Published:** 2024-08-27

**Authors:** P. Paribello, M. Manchia, U. Isayeva, R. Collu, P. Federica, M. Scherma, C. Pisanu, A. Meloni, C. C. Zai, D. Congiu, A. Squassina, W. Fratta, P. Fadda, B. Carpiniello

**Affiliations:** ^1^Department of Medical Sciences and Public Health, Unit of Clinical Psychiatry, University Hospital Agency of Cagliari, Cagliari, Italy; ^2^Department of Psychiatry, Institute of Medical Science, Laboratory Medicine and Pathobiology, University of Toronto, Toronto, Canada; ^3^Division of Neuroscience and Clinical Pharmacology, Department of Biomedical Sciences, University of Cagliari, Cagliari, Italy; ^4^Tanenbaum Centre for Pharmacogenetics, Campbell Family Mental Health Research Institute, Centre for Addiction and Mental Health, Toronto, Canada; ^5^Centre of Excellence “Neurobiology of Dependence”, University of Cagliari, Cagliari, Italy

## Abstract

**Introduction:**

Psychotic disorders present a significant lifetime risk for suicide. Past estimates suggest that up to 25-50% of individuals with schizophrenia (SCZ) may attempt suicide during their lifetime. A growing body of literature indicates that the level of cognitive performances may be associated with a differing level of lifetime suicide attempts, albeit inconsistently depending on the diagnostic category and study setting. However, the vast majority of the literature in the field is composed of cross-sectional studies, limiting the overall interpretation of the available evidence.

**Objectives:**

In the present study, we probed the possible association of BDNF plasma levels, cognitive functions assessed through the Brief Assessment of Cognition in Schizophrenia (BACS) and lifetime suicide ideation and/or attempts (LSI+LSA). More specifically, we tested whether such association would persist during the 2 years follow-up divided in 5 different timepoints at 6-month intervals, if present.

**Methods:**

The present study represents a secondary analysis of a previously described cohort (Manchia et al. Brain Sci. 2022 Dec 4;12(12):1666). The sample comprised 105 subjects with SZC or schizoaffective disorder. We employed the 1) Wilcoxon test for non-parametric data 2) linear modelling to test the possible association of BACS-defined cognitive task performances with LSI+LSA. We also investigated if either BDNF plasma levels or four tested BDNF SNP genes would mediate this association.

**Results:**

From a total of 105 subjects, data relevant to the analysis were available for 89 subjects. We observed a significant association between BACS-Letter fluency task (BACS-LF) with LSI+LSA, persisting even when adjusting for gender, duration of untreated psychosis, total Positive and Negative Syndrome Scale score, age, chlorpromazine equivalents of antipsychotic therapy and for the effect of time. The association remained significant even when adjusting with the Bonferroni-Holms method for multiple comparisons (p=0.002). No association was found either for BDNF plasma levels or the tested BDNF genes for the tested outcomes.

**Conclusions:**

In our sample, higher BACS-LF performances appeared to be associated with a higher lifetime risk of LSI+LSA. This report adds to the previous literature suggesting that different cognitive performance levels may represent one of the many chronic risk factors associated with LSI+LSA, and that may ultimately complexly interact with more proximal ones.

**Disclosure of Interest:**

None Declared

